# Autoimmune Diseases and Their Manifestations on Oral Cavity: Diagnosis and Clinical Management

**DOI:** 10.1155/2018/6061825

**Published:** 2018-05-27

**Authors:** Matteo Saccucci, Gabriele Di Carlo, Maurizio Bossù, Francesca Giovarruscio, Alessandro Salucci, Antonella Polimeni

**Affiliations:** Department of Oral and Maxillo-Facial Sciences, Sapienza University of Rome, Viale Regina Elena 287a, 00161 Rome, Italy

## Abstract

Oral signs are frequently the first manifestation of autoimmune diseases. For this reason, dentists play an important role in the detection of emerging autoimmune pathologies. Indeed, an early diagnosis can play a decisive role in improving the quality of treatment strategies as well as quality of life. This can be obtained thanks to specific knowledge of oral manifestations of autoimmune diseases. This review is aimed at describing oral presentations, diagnosis, and treatment strategies for systemic lupus erythematosus, Sjögren syndrome, pemphigus vulgaris, mucous membrane pemphigoid, and Behcet disease.

## 1. Introduction

Increasing evidence is emerging for a steady rise of autoimmune diseases in the last decades [1]. Indeed, the growth in autoimmune diseases equals the surge in allergic and cancer pathology; on the other hand, infections are shown to be less frequent in the Western societies [2]. Oral manifestations of autoimmune disease are frequently the primary sign of autoimmune diseases [3]. The dentists can therefore play a pivotal role in the detection and during the following multidisciplinary treatment. Precise and early diagnosis increases the efficiency and efficacy of treatment strategy [4–6]. Therefore, the goal of our review is to present the most common autoimmune diseases that show the first oral clinical signs and symptoms which are a manifestation of the general clinical disease. Our review is presenting details over systemic lupus erythematosus, Sjögren syndrome, pemphigus vulgaris, mucous membrane pemphigoid, and Behcet disease. Every single paragraph reviews the general conditions, and in the second part, we discuss the diagnosis and treatment strategies.

## 2. Systemic Lupus Erythematosus

Systemic lupus erythematosus (SLE) is a severe and chronic autoimmune inflammatory disease of unknown etiopathogenesis and various clinical presentations. SLE mainly affects women 8 times more likely than men. The worldwide prevalence of SLE ranges between 12 and 50 per 100,000, depending on location and ethnicity [7].

SLE is usually a chronic and progressive disease whose dormancy and progress are fairly regular and in sequence. There are cellular and cell-mediated processes involved in the SLE, even though it has been speculated that the primary involvement is mainly due to cell-mediated immunity and consequential humoral involvement [8]. The immune complex deposits in different organs triggering an inflammatory reaction that leads to organ functional impairment typical of the disease. In the pathogenesis of SLE, the activation of type I IFN pathways, B and T cell dysfunction, and presence of antinuclear antibodies were demonstrated [9]. Anti-DNA antibodies (deoxyribonucleic acid, antinuclear antibodies) are found in the patients' serum. The proliferation of these antibodies is supported by oestrogens. In some cases, there have been signs of antilymphocyte antibodies. The etiopathogenesis of SLE takes also into account genetic factors [8, 9].

Skin damage is the typical clinical sign of SLE, and it has been recorded in 85% of the cases [8]. Symptoms can vary from simple circular skin lesions to multiorgan impairment, potentially fatal. The most recurrent skin lesion is severe erythema on the surface of the skin exposed to light; also, oral discoid lesions are one of the more prevalent presentations of the disease. The so-called malar rash (or butterfly rush) is located on the nose and cheeks, and the erythema is found also on the finger tips. The healing process of these lesions which present a central scar and area around shows recrudescence very often. In SLE, we have involvement of joints, skin, muscles, eyes, lungs, central nervous system, and kidneys. At the joint level, arthralgia and arthritis are frequently associated with the advancement of SLE. The arthralgia has an asymmetric presentation and migratory behaviour [7]. The topography of joint manifestations is very wide. Indeed, it can interest any articular surface mimicking the rheumatoid arthritis. Deformities are generated by the inflammatory process of the tendons rather than degeneration [7]. At the skin level, purpuric manifestations and vitiligo can also be observed [8]. Lesions of retinae, as vasculitis, may injury the nerve fibres causing impairment or loss of vision. Renal disease or lupus nephritis is a grave complication of SLE that affects 30% of patients [10, 11]. The classical clinical manifestation is represented by a regular round or slightly red irregular area. This can be characterized by atrophy or the presence of ulceration. The red area is characterized by typical white radiating striae and telangiectasia. These signs may resemble those of lichen planus, despite the lack of symmetry. Although the oral condition is not major, petechial lesion and gingival bleeding such as desquamative gingivitis, marginal gingivitis, or erosive mucosal lesions have been reported in up to 40% of patients and may indicate serious thrombocytopenia. Many SLE patients may present at the same time Sjögren syndrome [8, 9, 12, 13].

### 2.1. Diagnosis

SLE diagnosis is based on a multiple-organ condition and the study of antinuclear antibodies at a serum level. The so-called LE cells can be detected in the blood stream. LE cells are mature neutrophils that have swallowed spherical inclusions produced by nuclear components and other cellular elements [8]. Lupus lesions can be confused with erythema multiforme lesions, lichen planus, and vesiculobullous lesions [7]. Moreover, the differential diagnosis has to include lichenoid reactions to dental fillings, traumatic or smoker's keratosis, and verrucous carcinoma [13]. The demonstration of intact adjacent tissues towards given lesions through histological and immunohistochemical confirmation is still the standard criterion for a definitive diagnosis [8, 12–14].

### 2.2. Treatment and Prognosis

SLE management is based on prevention, maintenance of states of remission and alleviation of symptoms, and reversal of inflammation [7, 8, 15, 16]. Salicylates and FANS are used in the less severe cases. There are other drugs used, such as hydroxychloroquine (an antimalarial), cortisones, and other immunosuppressants such as azathioprine and cyclophosphamide [8]. High- and medium-potency corticosteroids and calcineurin inhibitors are used as topical therapies for cutaneous manifestation [17]. Protection from sunlight is part of the strategy in order to avoid flare-ups of skin manifestations [7]. The prognosis is often good when the course of the disease is of an intermediate type and only few organs are involved. The disease can also be fatal in the case of kidney conditions with hypertension and rapid evolution towards kidney failure that leads to the patient's death [8, 18–20].

## 3. Sjögren Syndrome

Sjögren syndrome is an autoimmune disease affecting salivary and lacrimal glands and causing a reduction of the secretion activity due by lymphocytic infiltration and consequent destruction of the exocrine glands [8]. The lower production of saliva (hyposalivation) causes dryness in the mouth (xerostomia); the deficiency of tears causes xerophthalmia. Although the etiopathology of the Sjögren syndrome is still unknown, humoral- and cell-mediated immunity phenomena are involved in the process; as a matter of fact, increased activation of B cells followed by immune complex formation and autoantibody production plays important roles [21]. Genetic and environmental factors can also be part in the pathogenesis of the syndrome [13].

Sjögren syndrome affects 0.5–3% of the entire population and is predominant in women compared to men (9 : 1 ratio). Typically, Sjögren syndrome is detected around 50 years of age. It is important to underline that there are two characteristic surges: just after the menarche and after the menopause [13, 22, 23].

Some patients show clinical signs only confined to the mouth and eyes, while others present a more substantial autoimmune damage. 50% of the cases also have a different autoimmune condition, such as rheumatoid arthritis or systemic lupus erythematosus [8]. Damage to the glands without the evidence of other autoimmune issues is defined as primary Sjögren syndrome. The addition of an autoimmune disease is referred to as secondary Sjögren syndrome [13, 24]. The main signs of the syndrome are related to the oral cavity [8]. The xerostomia is responsible for creating different manifestations of SS at the level of oral cavity. Lack of saliva predisposes patients to develop tooth cavities. The lack of saliva facilitates the accumulation of plaque and their clearance. Edema and inflammations of the gingiva are frequent clinical signs. Moreover, a salivary flow decrease can develop opportunistic infections. *Candida* is often detected because the lack of lysozyme and immunoglobulins facilitates its development. Radfar et al. and Bayetto and Logan showed an association between *Candida* and the decreased stimulated salivary flow rate [25, 26]. The Sjögren syndrome affects both major and minor salivary glands. 50% of the cases show an increase in volume, symmetrical on both sides, of the parotid glands. The histological appearance of the hypertrophic glands is characterized by the replacement of the gland tissue by the lymphocytes and the presence of epimyoepithelial islands [24].

In addition to oral symptoms, patients also present irritation and dryness of the eyes, caused by xerophthalmia, as well as by photophobia. Nearly 20% of the patients affected by Sjögren syndrome show signs of the Raynaud phenomenon, a condition that affects fingers and toes [8]. Finally, patients affected by this disease may have arthralgia, myalgia, and asthenia.

The conclusions of different epidemiological studies claim, although newer studies are required to confirm this, that genetical as well as environmental factors play a role in the pathogenesis of the diseases [27, 28]. The syndrome is often accompanied by lab data alteration. 90% of the patients result positive to the rheumatoid factor, an anti-IgG antibody in the patient's serum. There are also other autoantibodies such as anti-Sjögren A and anti-Sjögren B that can be found in these patients [8].

### 3.1. Diagnosis

The diagnosis of Sjögren syndrome is basically clinical, supported by oral presentation and laboratory investigations. During recent decades, many classification criteria have been elaborated with the purpose to provide useful guidance for diagnosis by clinicians. The classification made by Shiboski et al. is generally utilized and also endorsed by the American College of Rheumatology [29, 30].

The diagnosis of the syndrome can be confirmed when two out of three of the following conditions are identified: xerostomia, keratoconjunctivitis sicca, and rheumatoid arthritis or another autoimmune disease [8]. Measuring the salivary flow and carrying out a biopsy of the minor salivary glands are two basic diagnostic investigation tests to detect the syndrome [24]. Very often, the xerostomia generates secondary symptoms that can help the clinician to orientate the diagnosis. Indeed, difficulties to speech and metallic sensation in the mouth are characteristic of xerostomia, as well as burning sensation of the oral mucosae [24, 31].

The ophthalmologic test is necessary to detect keratoconjunctivitis sicca. The lacrimal flow is measured by means of special absorbing pads [8]. Damage to the corneas, instead, requires further specific analysis. In most cases, the disease has a chronic and benign progress; however, these patients are exposed to a high risk to develop more serious clinical autoimmune issues: lymphoma and Waldenström macroglobulinemia. Periodical check-ups are mandatory in order to control and prevent the risks [8, 32, 33].

### 3.2. Treatment and Prognosis

The treatment for the Sjögren syndrome is mainly clinical. The use of FANS has a beneficial effect on arthritis. In major cases, corticosteroid and immunosuppressive drugs may be needed. Xerostomia can be regulated by using saliva substitutes such as sprays/gel or by installing an air humidifier. Sugar-free chewing gums may be useful to alleviate the feeling of dryness in the oral cavity, as well as hyperstimulate the salivary production. Methylcellulose artificial tears can alleviate xerophthalmia. Very often, Sjögren syndrome is accompanied by candidiasis produced by *Candida albicans*. This will require antimycotic treatment [13, 33]. Salivary secretion can be increased by taking pilocarpine. At a dental level, teeth and gums must be protected from the collateral damage caused by xerostomia [8]. Intensive domiciliary as well as professional oral hygiene care is mandatory to avoid complications due to teeth decay or root canal inflammation [34].

## 4. Pemphigus Vulgaris

Pemphigus vulgaris is a chronic immunomediated disorder. This disease affects the skin and mucosa. Patients affected by pemphigus have immune globulin G autoantibody against desmosomal components like desmoglein-1 and desmoglein-3 [35]. This alters the properties of adhesion cell molecules, producing intraepithelial blisters between the Malpighian epitheliocytes. This phenomenon is called *acantolysis of suprabasilar cheratynocites* [8, 35].

Although epidemiologically there is no evidence of gender predilection, some studies reported a slight prevalence in women [13]. All ages can be affected, though the highest number of cases is observed in patients in their 40s and 50s [8, 13].

The etiology would seem to be linked to genetic and ethnic factors. The lesions seem to be triggered by different inputs like physical agents, viruses, hormones, drugs, and stress [8, 13].

In over 50% of cases, the first signs of the disease arise in the oral mucosa. Although there is no area predilection, the lesions could be located at the buccal mucosae, soft palate, lower lip, and tongue and, less frequently, at the gingiva [36]. Oral lesions can range from fairly superficial ulcers to small vesicles or blisters. In the oral cavity, the bubbles rapidly break, leaving a painful erosion producing burning sensation [13]. The size of the ulcers is extremely variable. It can be noticed that a detachment of a large area of the surface with the formation of blisters can occur by exerting a slight pressure on the epithelium of these patients. This phenomenon is referred to as the Nikolsky phenomenon [8].

Pemphigus skin lesions are subsequent to oral manifestations. They can arise as simple rashes to erosions, vesicles, blisters, or ulcers. Microscopic examination highlights superficial epithelial damage with the intact basal layer adhering to the basal membranes [8]

### 4.1. Diagnosis

The pemphigus can be easily confused with other disorders that present lesions like aphthae, lichen planus, candidiasis, and pemphigoid. Often, pemphigus is associated with other autoimmune clinical situations such as the Sjögren syndrome, rheumatoid arthritis, and systemic lupus erythematosus [37, 38]. As a matter of fact, clinical, histopathological, and, in particular, direct and indirect immunofluorescence is mandatory to perform an efficient differential diagnosis. Direct immunofluorescence is performed on the tissue and highlights the local cellular damage (visualization takes place through a special microscope that highlights the fluorescence inside the spinous layer). In the indirect type, the antibodies are detected in the patient's serum [8, 39].

### 4.2. Treatment and Prognosis

The pemphigus is a pathology that involves primarily dermatologists although dentists can play an important role in the early diagnosis of the disease as well as in the management of oral manifestations. The treatment involves the administration of high-dose corticosteroids. In addition to these, immunosuppressive drugs such as azathioprine, cyclophosphamide, cyclosporine, and methotrexate are sometimes used. Recently, the use of rituximab has been proposed showing promising results [40, 41]. The titration of circulating antibodies is carried out to evaluate the progress of the disease. In fact, high antibody rates correspond to the most destructive phases of the disease. Their evaluation is also used to check the effectiveness of the treatment [8].

## 5. Mucous Membrane Pemphigoid

Mucous membrane pemphigoid (MMP) is a group of immune-mediated chronic blistering conditions. The oral mucosa is targeted as well as genital, conjunctival, and skin mucous membranes [8]. The autoantibodies mostly IgA and IgG are located, together with the C3 complement, on the mucosae as well as on epithelial basal membranes [35].

The most affected area is the gingiva, almost 94% of the cases [35], where the pemphigoid lesions give rise to a clinical condition called *desquamative gingivitis*. It has been said that desquamative gingivitis is not, per se, diagnostic. The lesions show as simple erythema or true ulcerations affecting both the fixed gingiva and the adherent gingiva. Very often, this lesion is confounded with periodontal disease.

However, lesions can also occur in other areas of the oral cavity including the palate, buccal mucosae, lips, tongue, and pharynx.

The symptoms associated with these conditions go from burning sensation and bleeding to masticatory impairment [35]. Pemphigoid blisters are less brittle than those seen in pemphigus and can remain intact in the oral cavity for up to 48 hours [8, 42].

### 5.1. Diagnosis

The diagnosis of mucous membrane pemphigoid is based on clinical and histological samples. The histologic examination shows the detachment of the epithelium from the underlying connective tissue. Direct immunofluorescence is diriment when there are doubtful histological samples showing a linear involvement at the level of the basal membrane. The immunofluorescence is particularly useful in the differential diagnosis with pemphigus and lichen as well as with periodontal disease and SLE. Epithelial degeneration is not observed; the connective tissue appears pervaded by an intense inflammatory infiltrate mainly consisting of plasma cells and eosinophils [8].

### 5.2. Treatment and Prognosis

The mucous membrane pemphigoid is a chronic disease that requires a continuous treatment strategy although the prognosis is benign. Sometimes, the lesions can only be localized to the gums; in other cases, the oral condition is wider. In less severe cases, the lesions can be treated by topical corticosteroid gel application although in some selected cases, it is coupled with dapsone (diaminodiphenyl sulfone). In the most severe forms, the treatment must be carried out systemically. Often, the pathology can be difficult to resolve, tending to respond rather late to therapy. It is crucial to monitor the presence of eye lesions to prevent ocular damage, such as injury to the cornea, conjunctiva, or eyelids [13, 35].

## 6. Behcet Disease

Behcet syndrome is an autoimmune, multisystemic disease of unknown etiology. It is typically characterized by at least two of the three key typical factors: oral ulcers, genital ulcers, and eye inflammation. Although its original definition is linked to dermatologic pathology, Behcet disease is often characterized by neurological and vascular involvement. It usually affects individuals in their 30s and shows no evidence of gender predilection. The greatest incidence of the disease is observed in Mediterranean and Asian populations with a marked prevalence in Turkey. The demonstration of an autoimmune genesis is given by the presence of antimucous autoantibodies, together with the association of the disease with the HLA configurations B5 and B51 [8, 43].

The mucocutaneous lesions are very often the first sign of the presence of Behcet syndrome. Their recognition is a key factor for early diagnosis, and they permit a more favourable prognosis [44]. The oral lesions are ulcers of the oral mucosae indistinguishable from the conventional aphthae of the oral mucosa. They are painful and characterized by cyclic presentation. They are localized at the lips, buccal mucosa, soft palate, and tongue. At the beginning, the lesion shows as an erythematous lesion, followed by an evolution in ulcers. Their dimensions can vary from few millimeters to centimeters [8, 44].

The genital ulcers are smaller and are located at the level of the scrotum, on the base of the penis, or on the labia majora.

Ocular lesions are present in 30–70% of the cases [43]. They show up as an initial form of photophobia, followed by uveitis and conjunctivitis. In some cases, they were found to be associated with glaucoma and cataract [43].

The skin lesions have a papular or pustular appearance and are mainly localized to the trunk or limbs.

### 6.1. Diagnosis

It has been said that there are not pathognomonic laboratory findings [43]. In order to diagnose the Behcet syndrome, according to the ISG criteria [45], at least two of the main features (oral, genital, or ocular lesions) must be present when another clinical explanation is excluded. Indeed, the differential diagnosis is a challenge considering that oral aphthous lesions are very common in the general population. Moreover, aphthous lesions are linked to HIV, Crohn's disease, sarcoidosis, and SLE, given that the dual-site-specific ulcerations seem to be the unique sign used to differentiate the Behcet syndrome from different pathologies cited above [43].

### 6.2. Treatment and Prognosis

The treatment of Behcet syndrome is based on the use of local and systemic cortisones per se or coupled with immunosuppressant drugs. The use of immunosuppressive drugs is justified by the lack of prevention of relapses due to the monocorticosteroid treatment strategy [43]. The main objective of Behcet syndrome patient care is to treat in time the oral mucocutaneous lesions in order to hinder the progression of the disease and to prevent the irreversible organ involvement in particular during the active phase [44]. Behcet syndrome could be fatal especially in the case of vascular involvement: aneurism rupture and thrombosis are the main causes of death.
